# Spermatogenic Activity and Sperm Traits in Post-Pubertal and Adult Tomcats (*Felis catus*): Implication of Intra-Male Variation in Sperm Size

**DOI:** 10.3390/cells10030624

**Published:** 2021-03-11

**Authors:** Eliana Pintus, Martin Kadlec, Barbora Karlasová, Marek Popelka, José Luis Ros-Santaella

**Affiliations:** 1 Department of Veterinary Sciences, Faculty of Agrobiology, Food and Natural Resources, Czech University of Life Sciences Prague, Kamýcká 129, 165 00 Praha 6-Suchdol, Czech Republic; kadlecmartin@af.czu.cz (M.K.); doskarovab@gmail.com (B.K.); 2 Veterinary Surgery Doskar, Podhorská 16, 150 00 Prague 5-Motol, Czech Republic; 3 Veterinary Clinic MyVet, Budějovická 81, 252 42 Jesenice, Czech Republic; mpopelka@veterinarni-klinika.com

**Keywords:** feline, intra-male variation, sexual maturity, spermatogenesis, spermatozoa, sperm morphometry, testis

## Abstract

Tomcats are considered to be adults at 1 year of age, although many reach sexual maturity at an earlier age. Nevertheless, we still know little about whether the spermatogenic activity and sperm quality of mature under one-year-old tomcats differ from those of tomcats that are over one-year-old. This study aims to evaluate the spermatogenic activity, sperm traits, and their relationships in mature tomcats at two different ages. Sixteen tomcats showing complete spermatogenesis and spermatozoa in their epididymal caudae were used and classified according to their age as post-pubertal (<1 year old) or adult (˃1 year old). Our results show that adult cats have higher epididymal sperm concentration and lower coefficient of variation in sperm head width and ellipticity than post-pubertal cats. However, they do not differ in their testicular and epididymal mass, spermatogenesis, and sperm traits such as motility, mitochondrial activity, morphology, morphometry, as well as plasma membrane, acrosome, and DNA integrity. Reduced intra-male variation of sperm head ellipticity is associated with higher testis mass, epididymis mass, and sperm concentration. Interestingly, low intra-male variation in sperm head size is associated with increased Sertoli cell function and reduced post-meiotic germ cell loss. These findings increase our knowledge about feline reproductive physiology and provide new insights into the functional significance of low intra-male variation in sperm size and shape in tomcats.

## 1. Introduction

The family Felidae hosts 41 species, most of which are endangered or face a serious decline in their population according to the International Union for Conservation of Nature [1,2]. The domestic cat (*Felis catus*) is the only, yet not fully, domesticated feline species that still shares many physiological and behavioral traits with its wild ancestors [3,4]. Similar to many of its wild relatives, the reproductive physiology of the domestic cat is characterized by induced ovulation in the queen and the frequent occurrence of teratospermia (i.e., ˃60% morphologically abnormal sperm cells in the ejaculate) in the tomcat. This biological proximity makes the domestic cat a valuable model for the optimization of assisted reproductive technologies, which are crucial for the success of breeding programs of other non-domestic feline species [4,5].

In tomcats, spermatogenesis lasts 46.8 days and can arise at 5 months of age, while sexual maturity is achieved between 7 and 12 months [6,7,8]. Although it has been reported that, starting from 7 months, more than 80% of cats show full spermatogenesis and epididymal sperm reservoirs [7], they are not considered to be adults until 1 year of age [9,10]. This implies that mature tomcats younger than 1-year-old are discarded in spite of their potential value for research or breeding purposes. Several studies have explored the changes that occur at sexual maturity in tomcats [7,11,12,13], but a comprehensive analysis about testicular and sperm parameters and their relationships is still lacking.

The aim of this study is to provide an exhaustive and detailed analysis of reproductive function in post-pubertal and adult tomcats. We assessed the impact of age on the testis mass, epididymis mass, spermatogenic activity, and epididymal sperm parameters (i.e., concentration, motility, DNA and plasma membrane integrity, acrosomal status, mitochondrial activity, morphology, and morphometry) and their relationships in 16 tomcats aged between 7 and 36 months. The results from this work increase our knowledge about the feline reproductive physiology and the time window for tomcats to be regarded as sperm donors for breeding or research purposes.

## 2. Materials and Methods

### 2.1. Reagents

All reagents were purchased from Sigma-Aldrich (Prague, Czech Republic).

### 2.2. Sample Collection and Sperm Recovery

Sixteen tomcats (from 7- to 36-months-old, body mass 4.28 ± 0.58 kg, mean ± SD) were used in the present study. All tomcats showed complete spermatogenesis and spermatozoa in their epididymal caudae. According to their age, the tomcats were classified as post-pubertal (9 males, <1 year old) or adult (7 males, ˃1 year old). Samples were collected from veterinary clinics after routine castration. Briefly, testes, epididymides, and part of the deferens ducts were placed in physiological solution (NaCl 0.9%, *w*/*v*) supplemented with gentamicin (50 µg/mL), stored at 5 °C, and processed within 24 h [14]. At the laboratory, testes and epididymides were separated and weighed to the nearest 0.01 g (HM-120, A&D Company, Tokyo, Japan). Then, one alternatively right or left epididymal cauda was placed in a glass Petri dish with 400 µL of previously warmed (38 °C) Hepes-TALP medium [14], supplemented with NaCl 85 mM (osmolality: ~400 mOsm/kg H_2_O, Osmomat 3000, Gonotec, Berlin, Germany). The epididymal cauda was finely cut using a sterile surgical blade and placed in an incubator at 38 °C for 10 min to allow the release of spermatozoa into the surrounding medium. Then, a sperm aliquot was fixed in 0.3% formaldehyde solution and sperm concentration was determined using a Bürker chamber. Then, the rest of the samples were diluted using Hepes-TALP medium to approximately 10–15 × 10^6^ spermatozoa/mL and incubated in a water bath at 38 °C for 20 min. At the end of incubation, sperm analyses were performed.

### 2.3. Sperm Analyses

#### 2.3.1. Motility

Sperm motility was evaluated through computer-assisted sperm analysis (CASA) (NIS-Elements, Nikon, Tokyo, Japan and Laboratory Imaging, Prague, Czech Republic), which consisted of an Eclipse E600 tri-ocular phase contrast microscope (Nikon, Tokyo, Japan), equipped with a warming stage set at 38 °C (Tokai Hit, Shizuoka, Japan), and a DMK 23UM021 digital camera (The Imaging Source, Bremen, Germany) [15,16,17]. Briefly, 5 µL of sperm samples was placed on a previously warmed (38 °C) Makler chamber (Sefi Medical Instruments, Haifa, Israel) and evaluated under a 10× negative phase-contrast objective. Several sperm kinetic parameters were assessed on at least 200 sperm cells: total motility (TM), progressive motility (PM), average path velocity (VAP, µm/s), curvilinear velocity (VCL, µm/s), straight-line velocity (VSL, µm/s), amplitude of lateral head displacement (ALH, µm), beat-cross frequency (BCF, Hz), linearity (LIN, %), and straightness (STR, %). The TM was defined as the percentage of sperm cells with VAP ≥ 10 µm/s, while PM was defined as the percentage of sperm cells with STR ≥ 80%. Sperm motility subpopulations were determined by cluster analysis (see statistical analysis). The standard parameter settings were as follows: frames per second, 60; minimum frames acquired per spermatozoon, 31; number of fields acquired, 6.

#### 2.3.2. Plasma Membrane Integrity and Acrosomal Status

For the assessment of plasma membrane integrity, 10 µL of sperm sample was placed in a staining solution composed of 2 μL propidium iodide (0.5 mg/mL in phosphate buffered saline solution, PBS), 2 μL carboxyfluorescein diacetate (0.46 mg/mL in dimethyl sulfoxide, DMSO), 1 μL formaldehyde (0.3%, *v/v* in PBS), and 85 µL of PBS. The osmolality of PBS was adjusted to ~400 mOsm/kg H_2_O by adding NaCl 85 mM. Then, the sample was incubated for 10 min at 38 °C in the dark [18]. After incubation, a subsample (10 μL) was placed on a clean microscope slide, covered with a coverslip, and evaluated under epifluorescence microscopy (40× objective, Eclipse E600, Nikon, Tokyo, Japan). Sperm heads that appeared entirely green were classified as being intact, while those that were partially or entirely red were classified as damaged. Acrosomal status was evaluated using fluorescein isothiocyanate-peanut agglutinin (FITC-PNA). Briefly, 10 µL of sperm sample was incubated with a staining solution composed of 3 µL FITC-PNA (200 µg/mL in PBS), 1 μL formaldehyde (0.3%, *v/v* in PBS), and 86 µL PBS for 10 min at 38 °C, in the dark. Then, 10 µL was placed on a clean microscope slide, covered with a coverslip, and evaluated under the same epifluorescence microscope used for the assessment of plasma membrane integrity. Sperm cells were classified as having an intact (no fluorescence over the entire acrosomal region) or damaged (green fluorescence over the acrosomal region) acrosome. For both analyses, 200 sperm cells were evaluated per each sample.

#### 2.3.3. Mitochondrial Activity

Mitochondrial status was evaluated using rhodamine 123 and propidium iodide, based on the protocol previously described [19], with some modifications. Briefly, 25 µL of sperm samples were incubated with 0.37 µL rhodamine 123 (5 mg/mL in DMSO), 2.5 µL propidium iodide (0.5 mg/mL in PBS), and 100 µL PBS for 15 min at 38 °C, in the dark. Then, samples were centrifuged at 500× *g* for 5 min, the supernatant was removed, and the sperm pellet was resuspended in PBS. Afterwards, 200 spermatozoa were evaluated using epifluorescence microscopy (40× objective). The spermatozoa showing bright green fluorescence over the midpiece were considered to have active mitochondria; those having low or no fluorescence over the midpiece were considered to have no active mitochondria.

#### 2.3.4. DNA Integrity

Sperm DNA was evaluated, as previously described by Thuwanut et al. [20]. Briefly, 10 µL of sperm sample were smeared on a microscope slide and air-dried. Then, samples were fixed in freshly prepared Carnoy’s solution (methanol:glacial acetic acid, 3:1, *v/v*) for 24 h, at room temperature. After that, samples were air-dried and stained for 5 min at room temperature, in the dark, with 50 µL of a staining solution composed of 100 µL acridine orange (1% in ultrapure water, *w*/*v*), 400 µL citric acid 0.1 M, and 25 µL Na_2_HPO_4_ 7H_2_O 0.3 M. Two hundred spermatozoa were evaluated under epifluorescence microscopy (40× objective); the sperm cells showing green fluorescence over the head were considered to have intact DNA, while those showing yellow or orange fluorescence over the head were considered to have damaged DNA.

#### 2.3.5. Morphometry and Morphology

**Figure 1:**
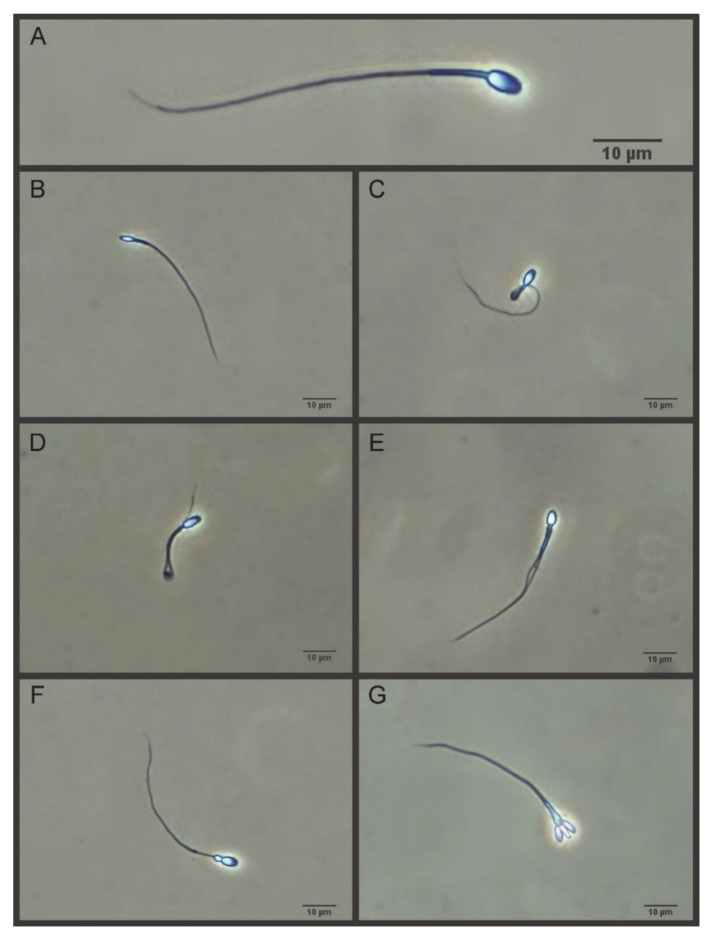
Sperm morphology in the tomcat. (**A**) Normal spermatozoon; (**B**) Head defect; (**C**) Midpiece defect; (**D**) Tail defect; (**E**–**G**) Multiple defects (40× phase-contrast objective).

Sperm morphology and morphometry were assessed, as previously reported [21,22] with some modifications. Sperm samples were fixed with a glutaraldehyde-PBS solution (final glutaraldehyde concentration of 0.25%, *v*/*v*), and then smeared on a slide. The smears were air-dried for at least 24 h, and then immersed in PBS for 10 min and mounted by sealing the edges of the coverslip (24 × 60 mm) with dibutyl phthalate xylene (DPX). Sperm pictures were taken using a digital camera (Digital Sight DSFi1, Nikon, Tokyo, Japan) under phase-contrast microscopy (Eclipse E600, 40× objective, Nikon, Tokyo, Japan). The resolution of the pictures was 2560 × 1920 pixels (TIFF format). The pixel size was 0.14 µm in the horizontal and vertical axes. Twenty-five sperm per sample were measured by ImageJ software (NIH, Bethesda, MD, USA) and the following morphometry parameters were determined: head length, head width, head perimeter, head area, head ellipticity (head length/head width), proximal midpiece width, midpiece length, principal piece length, terminal piece length, flagellum length, sperm length, and flagellum volume. For the assessment of sperm morphology, 200 cells were evaluated under phase contrast microscopy (40× objective) and classified as normal (sperm without any abnormality) or abnormal (head defects, midpiece defects, tail defects, proximal droplets, and multiple defects) sperm (Figure 1).

### 2.4. Spermatogenesis Assessment

**Figure 2:**
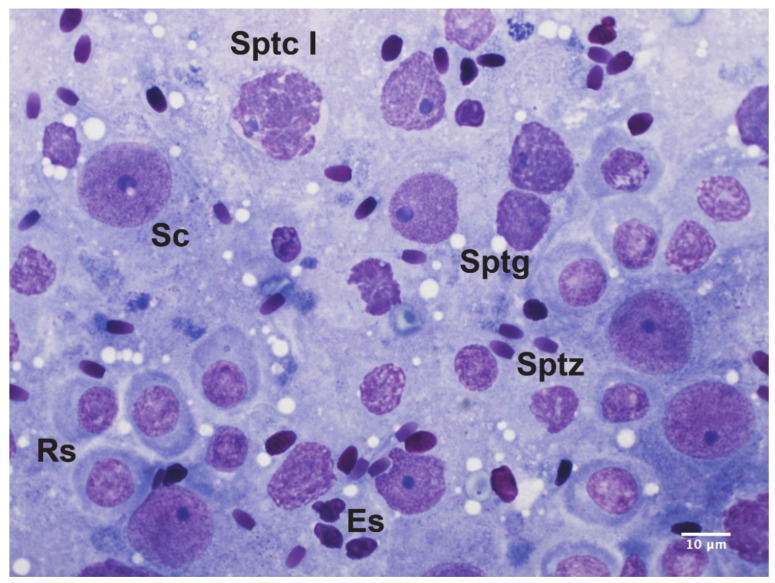
Spermatogenic and Sertoli cells in the tomcat. Sc, Sertoli cell; Sptg, spermatogonium; Sptc I, primary spermatocyte; Rs, round spermatid; Es, elongated spermatid; Sptz, spermatozoon. Hemacolor stain (100× bright-field objective).

Testicular cytology was performed on testicular imprints, as previously described [23]. Briefly, each testis was sectioned on the longitudinal plane and each side was gently laid on a clean microscope slide. Smears were air-dried for at least 24 h and stained by Hemacolor, as previously described [24]. Then, smears were mounted and evaluated under a 100× objective using bright-field microscopy (Eclipse E600, Nikon, Tokyo, Japan). At least 200 spermatogenic and Sertoli cells were counted per smear and classified according to the morphological criteria previously described in tomcat’s testicular cytology [23,25] (Figure 2). The following spermatogenic indices were calculated, as previously described by Ros-Santaella et al. [26]: Sertoli cell index (SEI), which is the percentage of Sertoli cells per total germ cells; spermatozoa–Sertoli index (SSEI), which is the ratio of sperm cells to Sertoli cells; meiotic index (MI), which is the ratio of round spermatids to primary spermatocytes and estimates the meiotic germ cell loss; ratio of elongated spermatids to round spermatids (ES/RS), which estimates the post-meiotic germ cell loss; ratio of elongated spermatids to total germ cells (ES/GC), which estimates the overall germ cell loss; ratio of round spermatids to Sertoli cells (RS/SC) and the ratio of elongated spermatids to Sertoli cells (ES/SC), which both estimate the Sertoli cell functionality; and the ratio of total germ cells to Sertoli cells (GC/SC), which estimates the workload capacity of Sertoli cells.

### 2.5. Statistical Analysis

Statistical analyses were performed using the SPSS 20.0 statistical software (IBM Inc., Chicago, IL, USA). The Shapiro–Wilk and Levene tests were used to check for normal distribution and homogeneity of variance, respectively. For determining sperm motility subpopulations, we used two kinetic parameters that define sperm average velocity (i.e., VAP) and trajectory straightness (i.e., STR). The number of clusters was automatically determined by the two-step cluster component using the Euclidean distance measure and Schwarz’s Bayesian criterion (BIC). After that, the number of clusters previously obtained was used to set up the K-Means cluster analysis by using the iteration and classification method. An independent sample *t*-test or the Mann–Whitney U test was used to check for differences between post-pubertal and adult sexually mature tomcats for data that were normally or not normally distributed, respectively. Two-tailed Pearson or Spearman rank correlations were applied to assess the relationship between parameters for data that were normally or not normally distributed, respectively. The *p* values < 0.05 were considered to be statistically significant.

## 3. Results

On average, sexually mature tomcats showed body mass ˃ 4 kg, testis mass ˃ 1.2 g, and epididymis mass ˃ 0.27 g (Table 1). We did not find any significant difference in body mass, testicular, and epididymal mass between groups, although there was a trend for epididymis mass to be higher in adult cats than in post-pubertal cats (*p* = 0.071, Table 1).

All tomcats showed normal and full spermatogenic activity (Table 2). In both groups, round spermatids were the most frequent spermatogenic cells, whereas secondary spermatocytes were the rarest. There were no differences between groups neither in the percentages of spermatogenic cells or in the spermatogenic indices (*p* ˃ 0.05, Table 2).

**Figure 3:**
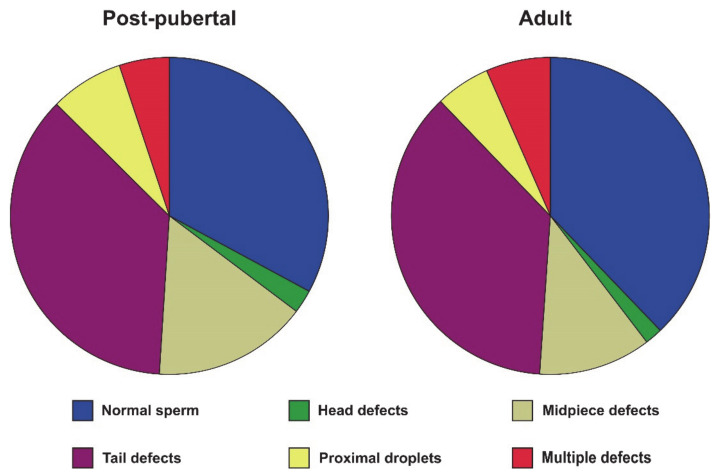
Sperm morphology in <1-year-old (post-pubertal, n = 9) and ˃1-year-old (adult, n = 7) sexually mature tomcats. No statistically significant differences were found between groups (*p* > 0.05).

Concerning sperm parameters, adult cats show more than two-fold epididymal sperm concentration than post-pubertal cats (*p* = 0.016, Table 3). There were no differences in the remaining sperm parameters, i.e., motility, morphology, mitochondrial activity, plasma membrane integrity, acrosomal status, and DNA integrity (*p* ˃ 0.05; Table 3 and Figure 3). Cluster analysis rendered four sperm subpopulations that, based on their kinetics, were classified as slow non-progressive (Sp1), rapid non-progressive (Sp2), medium progressive (Sp3), and rapid progressive (Sp4) (Table 4 and Supplementary Figure S1). Sperm subpopulations differed among them in most of the sperm kinematic parameters (Table 4). However, there were no significant differences between post-pubertal and adult tomcats in any sperm subpopulation (*p* > 0.05, Table 4).

The proportion of each sperm structure in relation to the total sperm length in tomcats (N = 16) was: head length, 8.56%; midpiece length, 13.45%; principal piece length, 70.71%; and terminal piece length 7.29%. In addition, sperm size did not differ between post-pubertal and adult cats (*p* ˃ 0.05, Table 5), whereas the intra-male variation in sperm head size did differ between groups (*p* < 0.05, Table 6). Interestingly, adult cats showed a lower intra-male coefficient of variation (CV) in sperm head width and ellipticity than post-pubertal adult cats (*p* < 0.05, Table 6).

**Figure 4:**
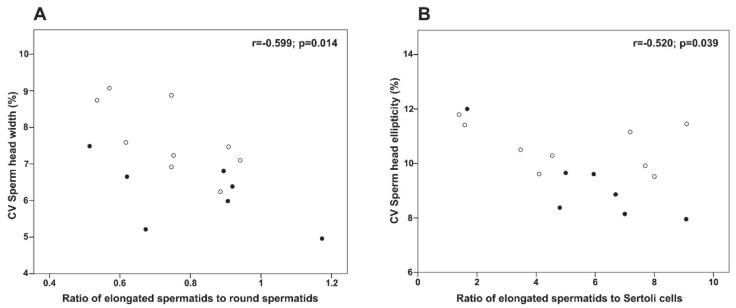
Relationships between intra-male coefficient of variation (CV) in sperm head size and spermatogenic function in sexually mature tomcats. Relationships between (**A**) CV of sperm head width and ratio of elongated spermatids to round spermatids (ES/RS; index of post-meiotic germ cell loss) and (**B**) CV of sperm head ellipticity and ratio of elongated spermatids to Sertoli cells (ES/SC; index of Sertoli cell functionality). White dots indicate <1-year-old sexually mature tomcats, while black dots indicate ˃1-year-old sexually mature tomcats.

We further tested the hypothesis that low intra-male variation in sperm head width and ellipticity was associated with improved spermatogenic activity and sperm quality (i.e., testis mass, epididymis mass, spermatogenic parameters, sperm morphology, velocity, and DNA integrity). Our results showed that a low CV of sperm head ellipticity was associated with high testis mass (r = −0.500, *p* = 0.049), epididymis mass (r = −0.499, *p* = 0.049), and sperm concentration (r = −0.631, *p* = 0.009). Interestingly, we also found that a low intra-male CV of sperm head size and shape was related to reduced germ loss during the spermiogenesis (ES/RS) and increased Sertoli cell function (ES/SC, *p* < 0.05, Figure 4).

There was no correlation between the CV of sperm head width and ellipticity with sperm morphology, velocity, motility subpopulations, DNA integrity, and the remaining spermatogenic parameters (*p* ˃ 0.05). However, it is worth noting that, although correlations were not statistically significant, a high CV of sperm head width was associated with a high incidence of sperm midpiece defects (r = 0.491, *p* = 0.053) and increased overall germ cell loss (ES/GC, r = −0.493, *p* = 0.052).

## 4. Discussion

Our findings show that tomcats can reach sexual maturity before 1 year of age and have very similar testicular and sperm parameters as those of over 1-year-old cats. There were, indeed, no differences between groups in testis mass, epididymis mass, spermatogenic activity, and most parameters of sperm functionality. Taken together, our findings suggest that epididymal sperm samples from sexually mature tomcats might be used for research or breeding purposes even if they are younger than 1 year of age. However, over one-year-old tomcats show higher epididymal sperm concentration and lower intra-male variation in sperm head size and shape as compared with those of under one-year-old tomcats. It remains to be investigated whether ejaculate’s traits differ between age groups. Overall, the values of body mass, testis mass, and epididymis mass are similar to the values previously reported by other authors [6,7]. Similarly, on the one hand, we also found that round spermatids were the most abundant spermatogenic cells, while secondary spermatocytes were the most scarce in a tomcat’s testicular cytology [23,25]. This pattern is consistent across several species such as red deer (*Cervus elaphus*) [24], fallow deer (*Dama dama*) [26], alpaca (*Vicugna pacos*) [27], and gemsbok (*Oryx gazella*) [28]. On the other hand, in jaguars (*Panthera onca*), elongated spermatids are the most abundant spermatogenic cells [29]. The abundance of round spermatids is likely due to the fact that theoretically, four round spermatids arise during the meiosis from each primary spermatocyte. By contrast, secondary spermatocytes are rarely observed because of their short lifespan that usually does not exceed 24 h. We also found that, on average, 2.6 round spermatids arise from each primary spermatocyte, which indicates that more than 30% of germ cells are lost during meiosis, consistent with that reported by previous studies (2.8 in [6]; 2.6 in [30]). In other felids such as lions (*Panthera leo*, meiotic index 2.7, [31]), jaguars (*P. onca,* meiotic index 2.8, [32,33]), cougars (*Puma concolor*, meiotic index 3.0, [34]), and ocelots (*Leopardus pardalis*, meiotic index 2.9, [35]), the meiotic index is generally higher than that of tomcats. Although no data are available in feline species about the index of post-meiotic germ cell loss (elongated spermatids per round spermatid ratio), the value found in our study (i.e., 0.8) is relatively high as compared with other species (e.g., 0.5 in deer) [24,26]. According to our findings, one-third of germ cells are lost during meiosis, while around one-fifth are lost during spermiogenesis. Similarly, Blanco-Rodriguez found that apoptotic germ cell death is maximal at pre-meiotic and meiotic phases and minimal at the post-meiotic phase in the adult tomcat [36].

Our study also shows that sperm motility, membrane and DNA integrity, mitochondrial activity, morphology, and morphometry do not differ between post-pubertal and adult tomcats. In agreement with the study by de Sousa Barbosa et al. [10] in tomcats and previous studies in jaguars (*P. onca*) and leopards (*Panthera pardus*) [37,38], we found that sperm tail defects were more frequent than head defects. Unlike the round-shaped sperm head of big cats (Pantherinae), spermatozoa from small cats (Felinae) show elongated and oval-shaped heads [39]. However, the sperm head size found in our study is relatively small as compared with the values recently reported by de Sousa Barbosa et al. [10], while the length of the sperm flagellum and its parts are rather similar. These differences are probably linked to the protocols used for sample preparation and analysis (e.g., Rose Bengal stained vs. unstained samples). The coefficients of variation of sperm size found in our study are also smaller overall as compared with those reported by de Sousa Barbosa et al. [10], probably because of the different methodology used (e.g., staining and rinsing procedures). Interestingly, our findings reveal that over one-year-old sexually mature tomcats have higher epididymal sperm concentration than under one-year-old tomcats. The lower epididymal sperm concentration of post-pubertal tomcats is likely a consequence of the recent onset of spermatogenesis; as a result, the epididymal storage capacity is still partially filled. Moreover, the intra-male CV of the sperm head morphometry differs between age groups, being significantly lower in over one-year-old tomcats. Interestingly, low intra-male variability in sperm head width and ellipticity is associated with enhanced Sertoli cell function and reduced post-meiotic germ loss. Sertoli cells play a vital role during spermatogenesis since they constitute the blood–testis barrier, provide nutritional support to the germ cells, and remove apoptotic and degenerating cells [40]. To the best of our knowledge, this is the first study showing that low intra-male variability in sperm size is associated with enhanced spermatogenic function in a feline species, providing interesting insights into a relatively poorly investigated sperm trait. Moreover, tomcats whose spermatozoa were more homogeneous in their sperm head ellipticity have higher testis mass, epididymal mass, and sperm concentration. Similarly, in an intraspecific study in red deer, intra-male CV of sperm head width was negatively associated with testes mass [22]. Another explanation for the reduced intra-male variation can be linked to the fluid reabsorption along the epididymal tract, which might influence sperm morphometry. The expression of estrogen receptor α, for instance, is both age- and segment-specific in the epididymis of tomcats and drives fluid reabsorption in the transition along the epididymal tract [12]. More recently, Gutiérrez-Reinoso and García-Herreros found that sperm cells from normospermic tomcats decrease their head width and increase their head ellipticity as they move from the caput to the cauda of epididymis [41]. It is interesting to consider that, in tomcats, the sperm head size is also associated with the degree of chromatin condensation [42] and that the percentage of sperm cells with normally condensed chromatin increases from the caput to the cauda of epididymis [43]. Therefore, in addition to the high spermatogenic activity and Sertoli cell function found in this study, a more efficient fluid reabsorption or chromatin condensation may also reduce the intra-male variation in sperm head size. To elucidate the functional implications of reduced intra-male variation in sperm head morphometry, we tested the hypothesis that low intra-male variation in sperm head size and shape might be associated with high sperm velocity (that is, more hydrodynamic head shape) and low percentage of sperm head abnormalities or damaged DNA. We found that the intra-male variation in sperm head width and ellipticity was not associated with sperm velocity or with sperm head normal morphology and DNA integrity. Although not significant, we found a positive relationship between the intra-male variation in the sperm head width and midpiece defects, which might have testicular or epididymal origins [44]. In agreement with our findings, Vernocchi et al. [45] found that head morphometry of tomcat epididymal spermatozoa did not correlate with the DNA status. It remains to be tested whether the low intra-male variation in sperm head morphometry might influence the sperm fertilizing ability in tomcats. In rams (*Ovis aries*), for instance, Gravance et al. found that the sperm head morphometry among fertile rams was extremely homogeneous [46]. In a more recent study in red deer, Ros-Santaella et al. found that low intra-male variation in sperm head size and shape was related to high sperm velocity and normal morphology [22], which are both parameters associated with male fertility in this species [47].

## 5. Conclusions

Our results show that tomcats can reach sexual maturity before 1 year of age. There are no differences between under one-year-old and over one-year-old sexually mature tomcats in their spermatogenic function, sperm velocity, motility subpopulations, morphology, morphometry, as well as plasma membrane, acrosome, and DNA integrity. Overall, our results suggest that epididymal spermatozoa from sexually mature tomcats could be used for research or breeding purposes, even if they are younger than 1 year of age. However, adult cats show higher epididymal sperm concentration and spermatozoa that are more homogeneous in their head size and shape. Reduced intra-male variation of sperm head ellipticity is associated with high testis mass, epididymis mass, and epididymal concentration, but not with sperm velocity, head morphology, and DNA status. Moreover, low intra-male variation in sperm head morphometry is associated with high Sertoli cell function and reduced post-meiotic germ cell loss. The results from this work increase our knowledge about feline reproductive physiology and provide new insights into the functional significance of low intra-male variation in sperm size and shape in tomcats.

## Figures and Tables

**Table 1:** Body mass, testis mass, and epididymis mass in <1-year-old (post-pubertal, n = 9) and ˃1-year-old (adult, n = 7) sexually mature tomcats.

Parameter	Post-Pubertal	Adult	*p* Value
Body mass (kg)	4.28 ± 0.62	4.29 ± 0.56	0.980
Testis mass (g)	1.24 ± 0.19	1.49 ± 0.52	0.275
Epididymis mass (g)	0.27 ± 0.05	0.31 ± 0.06	0.071

Data are shown as mean ± SD.

**Table 2:** Percentage of spermatogenic cell subtypes and indices in <1-year-old (post-pubertal, n = 9) and ˃1-year-old (adult, n = 7) sexually mature tomcats.

Parameter	Post-Pubertal	Adult	*p* Value
*Spermatogenic subtypes (%)*			
Spermatogonia	1.61 ± 0.88	1.53 ± 0.85	0.870
Primary spermatocytes	15.34 ± 5.62	14.57 ± 4.46	0.918
Secondary spermatocytes	0.60 ± 0.52	0.60 ± 0.40	0.918
Round spermatids	35.21 ± 3.18	34.87 ± 4.89	0.870
Elongated spermatids	26.03 ± 4.75	27.67 ± 5.63	0.536
Spermatozoa	21.22 ± 5.61	20.75 ± 7.66	0.888
*Spermatogenic indices*			
SEI (%)	7.00 ± 4.65	5.92 ± 3.45	0.758
SSEI	4.46 ± 3.16	4.89 ± 3.38	0.799
MI	2.58 ± 0.94	2.53 ± 0.66	0.905
ES/RS	0.74 ± 0.15	0.81 ± 0.22	0.467
ES/GC	0.26 ± 0.05	0.28 ± 0.06	0.532
RS/SC	7.07 ± 3.79	7.24 ± 3.20	0.928
ES/SC	5.23 ± 2.86	5.74 ± 2.30	0.706
GC/SC	19.78 ± 10.41	21.07 ± 9.30	0.801

Data are shown as mean ± SD. SEI, Sertoli cell index; SSEI, spermatozoa–Sertoli index; MI, ratio of round spermatids to primary spermatocytes (meiotic germ cell loss); ES/RS, ratio of elongated spermatids to round spermatids (post-meiotic germ cell loss); ES/GC, ratio of elongated spermatids to total germ cells (overall germ cell loss); RS/SC, ratio of round spermatids to Sertoli cells (Sertoli cell functionality); ES/SC, ratio of elongated spermatids to Sertoli cells (Sertoli cell functionality); GC/SC, ratio of total germ cells to Sertoli cells (Sertoli cell workload capacity).

**Table 3:** Epididymal sperm parameters in <1-year-old (post-pubertal, n = 9) and ˃1-year-old (adult, n = 7) sexually mature tomcats.

Parameter	Post-Pubertal	Adult	*p* Value
Concentration (10^6^/mL)	40.60 ± 26.37	92.48 ± 36.85	0.016
Intact plasma membrane (%)	83.94 ± 4.97	80.79 ± 7.96	0.346
Intact acrosome (%)	87.50 ± 4.65	88.14 ± 6.24	0.816
Active mitochondria (%)	68.33 ± 9.87	62.86 ± 7.77	0.249
Intact DNA (%)	88.50 ± 12.14	92.00 ± 4.80	0.837
Total motility (%)	63.15 ± 18.74	58.19 ± 18.25	0.604
Progressive motility (%)	50.14 ± 15.01	47.33 ± 11.57	0.688
VAP (µm/s)	97.81 ± 32.65	99.04 ± 19.46	0.932
VCL (µm/s)	151.93 ± 34.14	158.74 ± 20.05	0.648
VSL (µm/s)	84.82 ± 25.22	87.39 ± 14.77	0.814
ALH (µm)	5.61 ± 1.43	5.69 ± 0.70	0.899
BCF (Hz)	15.96 ± 3.05	16.00 ± 1.98	0.978
LIN (%)	52.03 ± 8.67	52.43 ± 6.39	0.536
STR (%)	85.12 ± 6.17	86.76 ± 6.64	0.918

VAP, average path velocity; VCL, curvilinear velocity; VSL, straight-line velocity; ALH, amplitude of lateral head displacement; BCF, beat-cross frequency; LIN, linearity; STR, straightness. Data are shown as mean ± SD.

**Table 4:** Epididymal sperm motility subpopulations in <1-year-old (post-pubertal, n = 9) and ˃1-year-old (adult, n = 7) sexually mature tomcats.

Parameter	Sp4	Sp3	Sp2	Sp1
VAP (µm/s)	138.07 ± 18.16 ^b^	76.11 ± 23.87 ^c^	177.10 ± 27.62 ^a^	6.09 ± 7.17 ^d^
VCL (µm/s)	201.23 ± 33.37 ^b^	136.34 ± 37.73 ^c^	235.22 ± 38.52 ^a^	20.54 ± 21.96 ^d^
VSL (µm/s)	126.64 ± 21.83 ^a^	70.25 ± 23.57 ^c^	107.71 ± 59.21 ^b^	3.48 ± 5.32 ^d^
ALH (µm)	7.28 ± 1.48 ^b^	4.51 ± 1.67 ^c^	9.37 ± 1.95 ^a^	1.02 ± 1.35 ^d^
BCF (Hz)	19.48 ± 7.67 ^b^	14.90 ± 7.86 ^c^	20.87 ± 4.96 ^a^	6.79 ± 3.36 ^d^
LIN (%)	64.01 ± 12.20 ^a^	51.87 ± 12.79 ^b^	45.31 ± 23.62 ^c^	14.15 ± 11.64 ^d^
STR (%)	91.63 ± 9.43 ^a^	92.42 ± 10.27 ^a^	58.45 ± 27.70 ^b^	44.11 ± 25.22 ^c^
Post-pubertal (%)	25.64 ± 15.45	21.38 ± 10.37	7.36 ± 11.48	45.61 ± 21.60
Adult (%)	24.40 ± 11.17	21.31 ± 10.37	4.48 ± 8.89	49.80 ± 16.84

Sp4, rapid progressive; Sp3, medium progressive; Sp2, rapid-non progressive; Sp1, slow non-progressive; VAP, average path velocity; VCL, curvilinear velocity; VSL, straight-line velocity; ALH, amplitude of lateral head displacement; BCF, beat-cross frequency; LIN, linearity; STR, straightness. Different superscripts within the same row indicate differences (*p* < 0.05). There were no differences between post-pubertal and adult cats within each sperm subpopulation. Data are shown as mean ± SD.

**Table 5:** Epididymal sperm morphometry in <1-year-old (post-pubertal, n = 9) and ˃1-year-old (adult, n = 7) sexually mature tomcats.

Parameter	Post-Pubertal	Adult	*p* Value
Head length (µm)	4.86 ± 0.26	4.97 ± 0.29	0.436
Head width (µm)	2.47 ± 0.10	2.42 ± 0.17	0.483
Head perimeter (µm)	11.84 ± 0.49	11.97 ± 0.53	0.598
Head area (µm^2^)	9.44 ± 0.65	9.46 ± 0.80	0.952
Head ellipticity	1.98 ± 0.13	2.07 ± 0.20	0.307
^§^Midpiece width (µm)	0.69 ± 0.04	0.69 ± 0.03	0.939
Midpiece length (µm)	7.67 ± 0.18	7.79 ± 0.24	0.268
Principal piece length (µm)	40.99 ± 1.59	40.06 ± 1.48	0.253
Terminal piece length (µm)	4.20 ± 0.57	4.16 ± 0.74	0.919
Flagellum length (µm)	52.85 ± 1.66	52.01 ± 1.98	0.369
Sperm length (µm)	57.72 ± 1.64	56.98 ± 2.12	0.447
Flagellum volume (µm^3^)	6.64 ± 0.75	6.53 ± 0.52	0.749

Data are shown as mean ± SD. ^§^Midpiece width was measured at proximal side.

**Table 6:** Intra-male coefficient of variation of epididymal sperm morphometry in <1-year-old (post-pubertal, n = 9) and ˃1-year-old (adult, n = 7) sexually mature tomcats.

Parameter	Post-Pubertal	Adult	*p* Value
Head length (%)	7.00 ± 1.17	6.64 ± 1.05	0.535
Head width (%)	7.69 ± 0.98	6.21 ± 0.90	0.008
Head perimeter (%)	5.47 ± 0.92	5.24 ± 0.75	0.610
Head area (%)	10.21 ± 1.53	9.18 ± 0.91	0.139
Head ellipticity (%)	10.63 ± 0.85	9.23 ± 1.39	0.026
^§^Midpiece width (%)	10.58 ± 2.22	10.45 ± 1.61	0.901
Midpiece length (%)	3.89 ± 1.46	4.65 ± 1.83	0.369
Principal piece length (%)	3.56 ± 1.42	3.21 ± 0.51	1.000
Terminal piece length (%)	15.06 ± 3.78	16.23 ± 2.64	0.496
Flagellum length (%)	3.12 ± 1.36	2.55 ± 0.42	0.536
Sperm length (%)	2.99 ± 1.15	2.43 ± 0.34	0.408
Flagellum volume (%)	21.43 ± 4.93	21.21 ± 3.52	0.922

Data are shown as mean ± SD. ^§^Midpiece width was measured at proximal side.

## Data Availability

Full dataset is available as supplementary information file Table S1.

## Supplementary Materials

The following are available online at https://www.mdpi.com/2073-4409/10/3/624/s1, Table S1: Full dataset, Figure S1: Goodness of fit of cluster analysis.
