# Supplementary figures and images for: Spermatogenic Activity and Sperm Traits in Post-Pubertal and Adult Tomcats (Felis catus): Implication of Intra-Male Variation in Sperm Size
Source: Cells. 2021 Mar 11;10(3):624. doi: 10.3390/cells10030624 (PMC7998732; doi:10.3390/cells10030624)

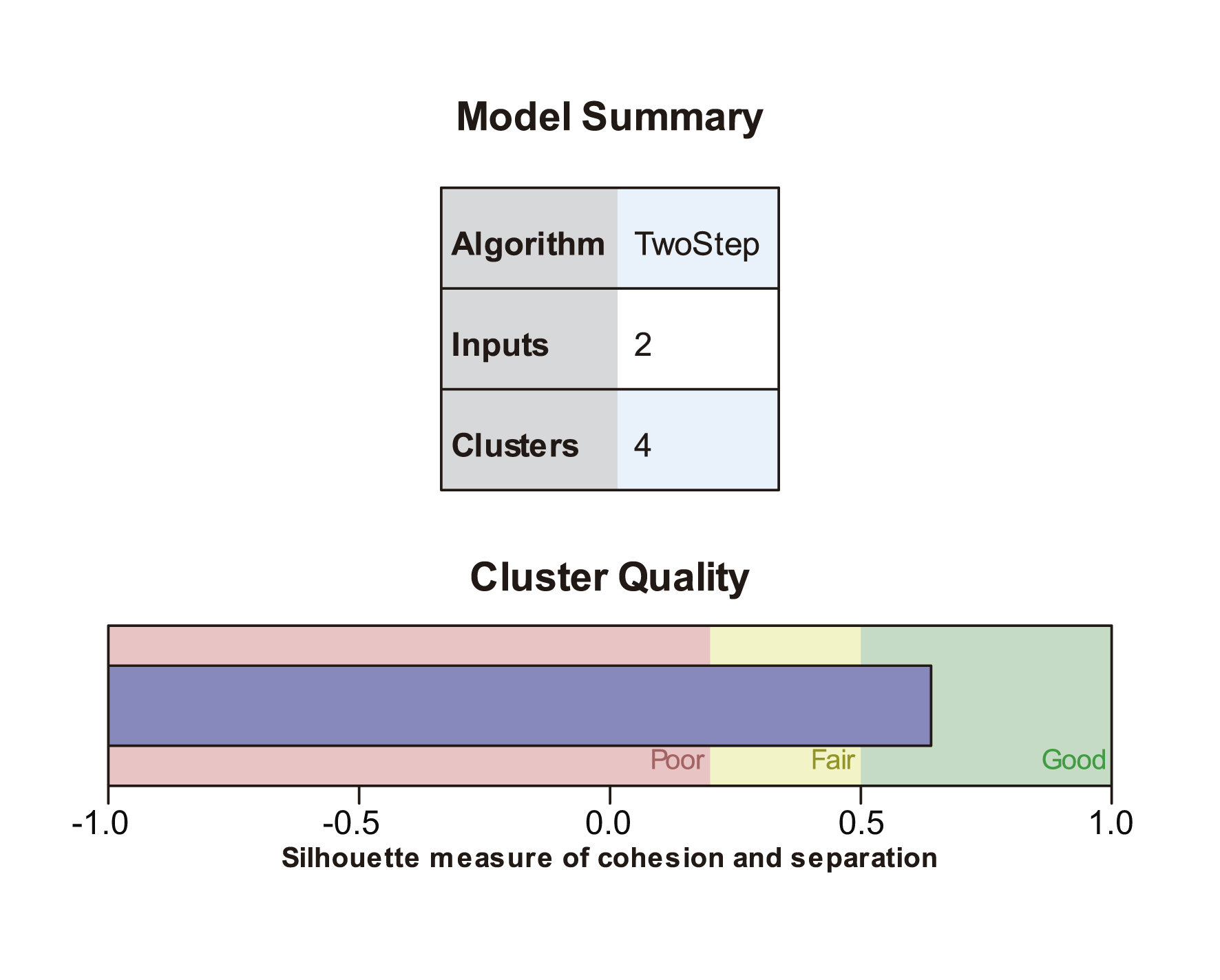

Supplement: Supplementary file 1 [file cells-10-00624-s001.zip › Figure S1.jpg]
